# Medication safety knowledge, attitude, and practice among hospital pharmacists in tertiary care hospitals in Saudi Arabia: a multi-center study

**DOI:** 10.1186/s13690-021-00616-1

**Published:** 2021-07-12

**Authors:** Azizah AL-Mutairi, Isamme AlFayyad, Youssef Altannir, Mohamad Al-Tannir

**Affiliations:** 1 Pharmacy Administration, King Fahad Medical City, P.O. Box. 59046, Riyadh, 11525 Kingdom of Saudi Arabia; 2 Research Center, King Fahad Medical City, P.O. Box. 59046, Riyadh, 11525 Kingdom of Saudi Arabia; 3 College of Medicine, AlFaisal University, P.O. Box 50927, Takhasusi Road, Riyadh, 11533 Kingdom of Saudi Arabia

**Keywords:** Medication safety, Pharmacovigilance, Adverse effect, Adverse drug reaction, Pharmacists, Saudi Arabia

## Abstract

**Background:**

Pharmacovigilance (PV) demarcates all actions involving the detection and prevention of adverse drug reactions (ADR) for marketed drugs. However, ADRs are considerably underreported worldwide and continue to be a major concern to health care systems. This study aims to assess the knowledge, attitude, and perception of hospital pharmacists regarding medication safety concerning PV and ADRs across multiple tertiary care centers around Saudi Arabia.

**Methods:**

This cross-sectional study was conducted between July 2019 and January 2020. Pharmacists working in the tertiary care centers of Riyadh City, Saudi Arabia were asked to participate in the study. A self-administered questionnaire was used to conduct this study, it consisted of: 63 questions out of which 19 questions were knowledge-based, 15 were attitude-based, and 29 were practice-based questions.

**Results:**

A total of 350 pharmacists were distributed and 289 agreed to participate, giving a response rate of 82.6%. Most pharmacists were aware of the concept of VP and its functions (96.5%) and (87.2%), respectively. Moreover, 90% said that ADR can be preventable and non-preventable. However, the findings revealed inadequate knowledge about the overall PV field, where the majority of the pharmacists failed to correctly answer questions related to independent ADRs treatment, Augmented drug reaction, the international location of ADR, and the World Health Organization “online database” for reporting ADRs. Moreover, incomplete and/or wrong answers were recorded for questions that included single or multiple correct answers. Regarding the participants” attitude, 96.9% were interested in ADR reporting, agreeing that ADR is important to enable safe drug usage. Although a general positive attitude was recorded, pharmacists have stated that the three main barriers that hinder reporting ADRs are: unavailability of information about ADRs, lack of awareness about the need to report ADRs, and lack of time. Concerning practice, 69.2% said they received training in ADRs reporting, and 70% have reported ADRs more than once a week.

**Conclusion:**

Surveyed pharmacists from Riyadh hospitals showed narrow knowledge of the PV field. However, a positive attitude and satisfactory practice was observed among pharmacists. These findings warrant the need for educational programs and an encouraging environment for ADR reporting to increase ADR reporting rates and support PV activities in Saudi Arabia.

**Supplementary Information:**

The online version contains supplementary material available at 10.1186/s13690-021-00616-1.

## Background

The provision of safe medications is a priority in health care systems; however, patients are inadvertently harmed by medication errors (ME) and adverse drug events (ADEs) [1]. Therefore, medication safety and pharmacovigilance are very essential in health care systems to ensure patient safety and remain to be a major concern to stakeholders, healthcare professionals, especially pharmacists, and inevitably, patients.

Higher incidents of ME and ADEs have been associated with increased morbidity and mortality across all age groups, prolonged or preventable hospitalization, and increased economic burden on health care systems and patients [2–4]. Clarifying ADEs, ME, and adverse drug reactions (ADRs) is a pivotal step that assists health care professionals in identifying illnesses, reporting incidents, and treating patients efficiently. The Institute of Medicine defines ADE as “an injury resulting from the use of a drug”; this includes harm caused by the drug (ADRs and overdoses) and harm from the use of the drug (including dose reductions and discontinuations of drug therapy)” [5]. The World Health Organization (WHO) defines ADR as “any response to a drug which is noxious and unintended and occurs at doses normally used in man for prophylaxis, diagnosis or therapy of disease or the modification of physiological function” [6]. Medication errors encompass all events that may occur at any stage of the medication process including prescribing, transcribing, dispensing, administering and monitoring, with or without patient harm [5].

Detection of the prevalence or incidence of ME, ADEs, and ADRs is difficult due to the different variables, including definitions and classification systems employed, hospital setting, availability of incident reporting systems, and events detection methods [7, 8]. Nevertheless, ADEs still occur and are expected to increase in the health care settings due to the development of new drug, the discovery of new indications of marketed drugs regarding different populations and aging, and the increasing use/misuse of drugs [9].

The term pharmacovigilance (PV) system has evolved after the thalidomide disaster in 1961 [10]. The PV system plays a principal role in ensuring medication safety through a systematic approach in collecting and analyzing ADEs associated with the use of marketed drugs, this allows for a more effective communication, lessening preventable harm incidents [11]. The WHO defines PV as the “science and activities related to the detection, assessment, understanding, and prevention of adverse effects or any other possible drug-related problems” [12]. Uppsala Monitoring Centre (UMC), located in Sweden, is the recognized body for the WHO Collaborating Centre for International Drug Monitoring. The UMC receives ADR reports from its members’ countries (PV national programs) to assess and communicate information in terms of drugs’ benefits, effectiveness, harm, and risks [13]. The Saudi Food and Drug Administration (SFDA) has established the National Pharmacovigilance Center in 2009 and became a member of the UMC to facilitate reporting of ADRs by all healthcare professionals and by pharmacists in particular [14].

The effectiveness and success of any PV system depends mainly on the participation of all healthcare practitioners, and the degree of reporting to, and the proper communication with the PV centers [15]. Recent studies have shown that ADRs are under-reported by healthcare practitioners, mainly in developing countries [16, 17]. Nonetheless, pharmacists play an essential role in preventing avoidable ADRs by carefully and systemically reviewing patients’ medical files and medical history, particularly drug-drug allergy and previous history of ADRs [18]. In 2017, the WHO launched the third Global Patient Safety initiative as ‘medication without harm’ to halve avoidable harm related to medication over 5 years [19]. The success of this ambitious target requires accurate data on the prevalence and consequence of ME, ADEs, and ADRs which depends on proper and systematic reporting to the PV centers by pharmacists.

Therefore, the assessment of knowledge, attitude and perceptions of pharmacists regarding medication safety is essential to support this initiative and to come up with the needed educational training that will result in an increased quality of healthcare services provided to patients by pharmacists.

In this study, we aim to assess the knowledge, attitude, and perception of hospital pharmacists on medication safety concerning adverse drug events and pharmacovigilance across multiple tertiary care centers around Saudi Arabia.

## Methods

### Study design and settings

Cross-sectional study design was conducted between July 2019 and January 2020 among hospital pharmacists working at tertiary care hospitals in Riyadh City, Saudi Arabia. The hospitals included public, private, and University medical hospitals that provide medical services across the five regions of Riyadh, namely (Central, North, South, East, and West regions).

### Sampling technique and sample size

Using a convenient sample technique, all pharmacists who were dealing with medications on a daily basis were invited to participate in the study. Pharmacists that were excluded from the study included those working in administrative positions, secondary and primary hospitals and community pharmacies. Considering a total number of 5000 hospital pharmacists, the online Raosoft sample size calculator estimated a minimum sample size of 289 hospital pharmacists to ensure a confidence level of 95% with a 5% margin of error assuming that 50% of hospital pharmacists’ express good knowledge of medication safety.

### Data collection

A self-administered questionnaire was used to assess medication safety knowledge, attitude, and practice among hospital pharmacists. The questionnaire was designed based on a previous study but was modified by local senior administrative pharmacists with long expertise in the hospital practice [9]. The questionnaire was administered in English since education in Saudi Arabia is English based. The reliability of the questionnaire was conducted with a pilot study of 30 pharmacists working in 3 different tertiary care centers, and the Cronbach alpha was 0.74.

The final survey consisted of four sections and included 63 questions; of which 19 questions were knowledge-based, 15 were attitude-based, and 29 were practice-based questions. In addition, the survey included 5 questions regarding pharmacists profile and another 4 questions about hospital descriptions. A detailed explanation of the questions with the correct answers can be found in Additional file 1 The questionnaire was distributed and handed to all pharmacists individually by assigned data collectors. The study objectives were explained to each pharmacist.

### Data analysis plan

Statistical analysis was performed using IBM SPSS software, version 23 (Armonk, NY: IBM Corp.). Descriptive analyses were used to present the results as percentages and frequencies. Confidence intervals for the pharmacists’ response to the questions were assessed.

## Results

### Sociodemographic results

A total of 350 questionnaires were distributed to pharmacists, where 289 participants completed the questionnaire and were included in the final data analysis with a response rate of 82.6%. The results revealed that 201 (69.6%) of pharmacists were female, 179 (61.9%) aged between 21 and 30 years, and 195 (67.5%) have > 5 years of experience. Pharm-D represented 69.9% of the participants and the majority 78.6% were staff pharmacists. The greatest number of the visited hospitals encompassed beds capacity more than 300 beds and 93.1% of the hospitals were Saudi Central Board for Accreditation of Healthcare Institutes (CBAHI) accredited. In addition, 86.5% had acquired an International Organization for Standardization (ISO) accreditation Table 1.

**Table 1:** Pharmacist’s characteristics and hospitals profile

Factor	n (%)
**Gender**	
Male	88 (30.4)
Female	201 (69.6)
**Age (years)**	
21–30	179 (61.9)
31–40	102 (35.3)
> 40	8 (2.8)
**Level of Education**	
Bachelor	91 (31.5)
Pharm-D	179 (61.9)
Master	19 (6.6)
**Professional Status**	
Pharmacist Assistant	20 (6.9)
Staff Pharmacist	227 (78.6)
Clinical Pharmacist	22 (7.6)
Senior Pharmacist	20 (6.9)
**Experience**	
< 5 Years	195 (67.5)
5–10 Years	74 (25.6)
> 10 Years	20 (6.9)
**Business Structure**	
Public	149 (51.6)
Private	60 (20.8)
University Medical Center (owns a university)	80 (27.6)
**Number of Beds**	
≤ 50	10 (3.5)
51–100	24 (8.3)
101–299	45 (15.6)
≥ 300	210 (72.6)
**Occupancy Rate**	
< 50%	0
50–65%	45 (15.6)
66–80%	55 (29.1)
> 80%	189 (65.3)
**Accreditation**^**a**^	
Saudi CBAHI Accredited	269 (93.1)
JCI Accredited	250 (86.5)
ISO Certified	70 (24.2)
Magnet Certified	100 (34.6)
Not Accredited or in process	20 (6.9)

^a^*CBAHI* Central Board for Accreditation of Healthcare Institutes, *JCI* Joint Commission International, *ISO* International Organization for Standardization

### Knowledge of hospital pharmacists concerning pharmacovigilance

The results pertaining to the pharmacists’ knowledge of pharmacovigilance revealed that 250 (86.5%) of the pharmacists included in the study correctly answered that “a side effect of a drug is an adverse drug reaction”, and 260 (82.1%) correctly answered that “an adverse drug reaction is preventable and non-preventable”. Furthermore, 96.5% of the participants correctly answered that “not all the drugs available in the market are safe” Table 2.

**Table 2:**
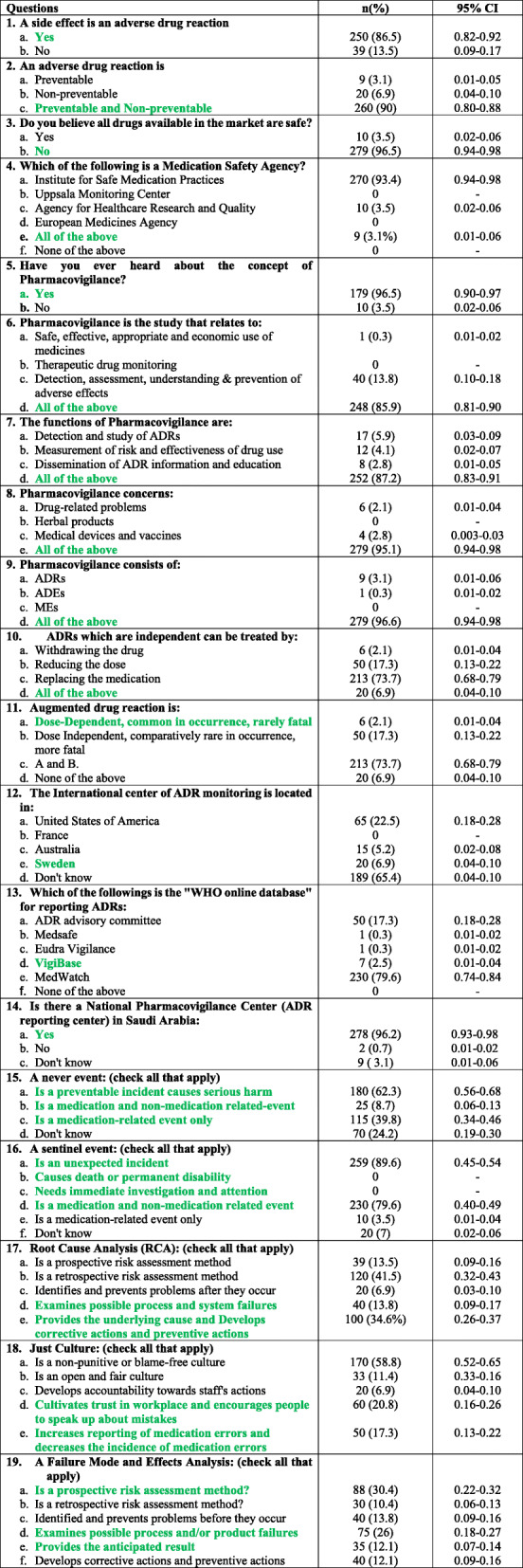
Knowledge of the hospital pharmacists concerning pharmacovigilance

^*^Answers in green are the correct answers to the questions

Remarkably, the vast majority of the pharmacists (93.4%) knew that “the Institute for Safe Medication Practices, the UMC, the European Medicines Agency, and the Agency for Healthcare Research and Quality” are medication safety agencies. Almost 97% of the respondents have heard about the concept of pharmacovigilance, and 87.2% pharmacists correctly knew the functions of pharmacovigilance. Moreover, 95.1% of the participant pharmacists correctly answered that pharmacovigilance concerns drug-related problems, herbal products, medical devices, and vaccines. However, only 2.1% of the respondents correctly knew that “an augmented drug reaction is dose-dependent, common in occurrence and rarely fatal” Table 2.

About 96.2% knew that there is a pharmacovigilance center in Saudi Arabia. Regarding the definition of a never event, 24.2% did not know the correct answer. In addition, in regards to the definition of a sentinel event, 89.6% of the pharmacists declared that it is “an unexpected incident”, 79.6% of them said, “it causes death or permanent disability”. Moreover, regarding the root cause analysis, 13.8% answered that it “examines possible process and system failures” and 34.6% answered that it “provides the underlying cause”, (both statements are correct answers). Concerning the “just culture” definition, 30.4% answered that “it cultivates trust in the workplace” and 17.3% attested “it increases the reporting of medication errors”, whereas 58.8% stated that “it is a non-punitive or blame-free culture”. In addition, in regard to the definition of Failure Mode and Effect Analysis, 30.4% of the participants reported that “it is a prospective assessment method”, 26% reported that “it examines possible process and system failures” and 12.1% reported that it “provides the underlying cause”. All three statements are the correct answers Table 2.

### Attitude of hospital pharmacists concerning pharmacovigilance

In response to the attitude questions raised in the questionnaire, 100% of the pharmacists agreed that the pharmacist should be the healthcare professional responsible for reporting ADRs in the hospital. However, 93.4% of the participant pharmacists supported “direct adverse drug reaction reporting by the patient instead of healthcare professionals”. All study participants agreed that “Adverse drug reaction reporting and monitoring system would benefit the patient” and that “pharmacists should be the ones to assist physicians in adverse drug reaction reporting”. However, 31.5% of the pharmacists attested “worrying about legal problems while thinking of adverse drug reactions”. Interestingly, 31.5% of the pharmacists believed that “reporting adverse drug reactions is a time-consuming activity with no outcome”. Nearly, 73% of the pharmacists believed that adverse drug reaction reporting should be made mandatory for a practicing pharmacist, and 97% of the pharmacists “are interested in participating in an adverse drug reaction reposting system”. All study participants believed that “there should be a national pharmacovigilance program” Table 3.

**Table 3:** Attitude of the hospital pharmacists concerning pharmacovigilance

Questions	n (%)	95% CI
**1. Which of the following health care professionals is/are responsible for ADRs reporting in your hospital?**		
a. Doctor	250 (86.5)	0.82–0.90
b. Pharmacist	289 (100)	1
c. Nurses	230 (79.6)	0.74–0.84
**2. Do you support “direct ADR reporting” by the patient instead of health care professionals?**		
a. Yes	270 (93.4)	0.94–0.98
b. No	19 (6.6)	0.04–0.10
**3. Do you think that ADR reporting and monitoring systems would benefit the patient?**		
a. Yes	289 (100)	1
b. No	0	–
**4. Do you think pharmacists are the ones to assist physicians in ADR reporting?**		
a. Yes	289 (100)	1
b. No	0	–
**5. Do you worry about legal problems while you think of ADR?**		
a. Yes	91 (31.5)	0.26–0.37
b. No	198 (68.5)	0.63–0.74
**6. Do you feel that ADR reporting is a time-consuming activity with no outcome?**		
a. Yes	91 (31.5)	0.26–0.37
b. No	198 (68.5)	0.63–0.74
**7. Do you think reporting is a professional obligation to you?**		
a. Yes	120 (41.5)	0.36–0.47
b. No	10 (3.5)	0.02–0.06
c. Don’t know	80 (27.7)	0.23–0.33
d. Perhaps	79 (27.3)	0.22–0.33
**8. Do you believe reporting should be made mandatory for practicing pharmacists?**		
a. Yes	200 (69.2)	0.63–0.74
b. No	79 (27.3)	0.22–0.33
c. Don’t know	10 (3.5)	0.02–0.06
**9. Are you interested in participating in an ADR reposting system?**		
a. Yes	280 (96.9)	0.94–0.99
b. No	9 (3.1)	0.01–0.06
**10. Do you think there should be a National Pharmacovigilance Program?**		
a. Yes	289 (100)	1
b. No	0	–
**11. If a National Pharmacovigilance Program is instituted, what is your expectation from it?**		
a. Someone from the center to coordinate with you.	273 (94.5)	0.91–0.97
b. Financial compensation for time and energy spent.	16 (5.5)	0.03–0.09
**12. ADR reporting is important in order to: (check all that apply)**		
a. Enable safe drugs to be used.	289 (100)	1
b. Measure the incidence of ADRs.	270 (93.4)	0.90–0.96
c. Identify previously unrecognized ADRs.	270 (93.4)	0.90–0.96
d. Identify factors that might predispose to an ADR.	270 (93.4)	0.90–0.96
e. Compare ADRs for drugs in similar therapeutic classes.	221 (76.5)	0.71–0.81
f. Compare ADRs of the same drug from different drug companies.	221 (76.5)	0.71–0.81
**13. In your opinion, pharmacist is encouraged to report ADRs when? (check all that apply)**		
a. The reaction is of a serious nature	289 (100)	1
b. The reaction is unusual	240 (83)	0.78–0.87
c. The reaction is to a new product	240 (83)	0.78–0.87
d. The reaction is not reported before a particular drug	240 (83)	0.78–0.87
e. The reaction is well recognized for a particular drug	159 (55)	0.49–0.61
**14. In your opinion, pharmacists may be discouraged to report ADRs when: (check all that apply)**		
a. Level of clinical knowledge makes it difficult to decide whether or not an ADR has occurred	18 (6.2)	0.03–0.10
b. Uncertain Association Between the drug and the adverse reaction	7 (2.4)	0.01–0.05
c. The ADR is too trivial to report	3 (1)	0.01–0.03
d. Concern that a report will generate extra work	3 (1)	0.01–0.03
e. ADR reporting form is not available when needed	2 (0.7)	0.01–0.02
f. Not Enough Information Available from the patient	280 (96.9)	0.94–0.99
g. Lack of time to fill in a report	230 (79.6)	0.74–0.84
h. Did not know how to report	17 (5.9)	0.03–0.09
i. Unaware of the need to report an ADR	280 (96.9)	0.94–0.99
j. Consider if the doctors’ responsibility	2 (0.7)	0.01–0.02
k. Fear of legal liability	0	–
**15. The perception of a safety culture revolves around which of the following statements:**		
a. Why waste our time on safety	0	–
b. We Do Something When We Have an incident	135 (46.7)	0.41–0.43
c. We have systems in place to manage all identified risks	6 (2.1)	0.01–0.04
d. We are always on the alert for risks that might emerge	7 (2.4)	0.01–0.05
e. Risk management is an integral part of everything that we do	141 (48.8)	0.43–0.55

Regarding the importance of reporting adverse drug reactions, 100% of the respondents believed that “it enables the safe use of drugs”, 93.8% reported that it “measures the incidence of adverse drug reactions”, and 93.4% believe that it helps “identify factors that might predispose to an adverse drug reaction”. 100% of the pharmacists reported that they are encouraged to report adverse drug reactions “when the reaction is of serious nature”. On the contrary, 7% reported that they are discouraged to report adverse drug reactions when there is an uncertain association between the drug and the adverse event. Besides, 48.8% of the pharmacists reported that regarding the safety culture, risk management is an integral part of their work Table 3.

### Practice of hospital pharmacists concerning pharmacovigilance

In regard to the practice of hospital pharmacists pertaining to pharmacovigilance, almost three-quarters of them declared that “all medication safety-related standards are applied in the hospital they work at”, and 100% of the respondents declared that the hospitals they work at have written policies and procedures about medication safety practices. Besides, 74.7% of the pharmacists declared that the respective hospitals have an established system for adverse drug reporting. The majority 96.5% declared having a medication safety committee or department in their respective working place, of whom 99.6% attested that the pharmacy chair overlooks the aforementioned committee. All pharmacists declared that “there is a standardized form for reporting adverse drug reactions in their hospital and it is accessible in the pharmacy. Slightly more than three-quarters of the participants indicated they have a work environment encourage reporting ADR, however, 48.1% reported they never encountered an ADR Table 4.

**Table 4:** Practice of the hospital pharmacists concerning pharmacovigilance

Questions	n (%)	95% CI
**1. Concerning the national accreditation Pharmacy chapter, are all medication safety-related standards applied in your hospital?**		
a. Yes	216 (74.7)	0.69–0.80
b. No	73 (25.3)	0.20–0.31
**2. Does your hospital have written policies and procedures on safe medication practice?**		
a) Yes	289 (100)	1
b) No	0	–
**3. Has your hospital established a defined system for adverse events reporting?**		
a) Yes	216 (74.7)	0.69–0.80
b) No	73 (25.3)	0.20–0.31
**4. Is there a “Medication Safety” or a “Safety Committee/Department” in your hospital?**		
a) Yes	279 (96.5)	0.94–0.98
b) No	10 (3.5)	0.02–0.06
**5. If yes, Is the pharmacist a member of the committee/department?**		
a. Yes	278 (99.6)	0.94–0.98
**6. If yes, does the pharmacist chair/oversee that committee/department?**		
a. Yes	278 (99.6)	0.94–0.98
**7. In your hospital, is there a standardized form for reporting ADRs?**		
a. Yes	289 (100)	1
b. No	0	–
**8. Is the reporting form available at your Pharmacy?**		
a. Yes	279 (96.5)	0.94–0.98
b. No	10 (3.5)	0.02–0.06
**9. Does your workplace encourage you to report any ADR?**		
a. Yes	220 (76.1)	0.71–.081
b. No	69 (23.9)	0.19–0.29
**10. Have you ever come across an ADR?**		
a. Yes	150 (51.9)	0.46–0.58
b. No	139 (48.1)	0.42–0.54
**11. In your hospital, ADRs are reported only when they are: (check all that apply)**		
a. Serious and life-threatening	279 (96.5)	0.94–0.98
b. Severe and cause disability	279 (96.5)	0.94–0.98
c. Mild and cause less inconvenience	282 (97.6)	0.95–0.99
**12. When an ADR is encountered in your hospital, it is reported to:**		
a. Patient	21 (7.3)	0.05–0.11
b. Prescriber	3 (1)	0.002–0.30
c. Drug Company	12 (4.2)	0.22–0.07
d. MOH	40 (13.8)	0.10–0.18
e. Saudi FDA	279 (96.5)	0.94–0.98
**13. How do you prefer to report ADRs to drug companies? (check all that apply)**		
a. Verbally inform the representative of the drug company on routine visits	2 (0.7)	0.01–0.02
b. Formal email/letter.	201 (69.6)	0.63–0.75
c. Phone call.	0	–
d. Via a National Pharmacovigilance Center.	279 (96.5)	0.94–0.98
**14. Have you attended any congress/ seminar on continuing educational program on safe medication practice issues in the last year?**		
a. Yes	49 (17)	0.13–0.22
b. No	240 (83)	0.78–0.87
**15. Have you ever had a course/attended a workshop about pharmacovigilance?**		
a. Yes	145 (50.2)	0.44–0.56
b. No	144 (49.8)	0.44–0.56
**16. Have you anytime read any article on prevention of ADRs?**		
a. Yes	250 (86.5)	0.82–0.90
b. No	39 (13.5)	0.10–0.18
**17. Have you ever been trained on how to report ADRs?**		
a. Yes	200 (69.2)	0.64–0.74
b. No	89 (30.8)	0.26–0.36
**18. Do you think Pharmacovigilance should be taught in detail to healthcare professionals?**		
a. Yes	280 (96.9)	0.94–0.98
b. No	9 (3.1)	0.01–0.06
**19. Do you think medication safety (ADR) programs should be included in the actual Pharmacy curriculum?**		
a. Yes	283 (97.9)	0.96–0.99
b. No	6 (3.1)	0.01–0.04
**20. Which of the following medication safety preventive measures are applied in your Pharmacy? (check all that apply)**		
a. Unit dose labeling	200 (69.2)	0.63–0.74
b. Unit dose labeling per patient	200 (69.2)	0.63–0.74
c. Look-A-Like / Sound-A-Like labeling	289 (100)	1
d. High Alert Medication labeling	279 (96.5)	0.94–0.98
e. Use of TALLman letters Avoidance of ambiguous nomenclature (abbreviations, trailing zeroes)	289 (100)	1
f. Bar-coding	189 (65.4)	0.60–0.71
g. Temperature monitoring	220 (76.1)	0.71–0.81
**21. In your Pharmacy, are there any staff educational sessions on medication safety best practices?**		
a. Yes	220 (76.1)	0.71–0.81
b. No	69 (23.9)	0.19–0.29
**22. When ADRs are reported, which of the following assessment methods are implemented in your hospital:**		
a. Root Cause Analyses (RCA)	0	–
b. Failure Mode and Effects Analysis	0	–
c. Causality Assessment tools	10 (3.5)	0.02–0.06
d. Severity Assessment tools	10 (3.5)	0.02–0.06
e. Classification Tools	9 (3.1)	0.01–0.06
f. All of the above	259 (89.6)	0.86–0.93
g. None of the above	1 (0.3)	0.0001–0.02
**23. Are analysis results reported to the Pharmacy & Therapeutics committee?**		
a. Yes	260 (90)	0.86–0.93
b. No	29 (10)	0.07–0.14
**24. How often are ADRs reported?**		
a. More than once a week	201 (70)	0.64–0.75
b. Once Month	70 (24.2)	0.19–0.30
c. A few times a year	16 (5.5)	0.03–0.09
d. Never	2 (0.7)	0.01–0.02
e. No answer	0	–
**25. Whether electronic and/or paper-based, are ADRs documented in the patient medical record?**		
a. Yes	189 (65.4)	0.60–0.71
b. No	100 (34.6)	0.29–0.40
**26. If yes, is there an alerting system, such as pop-up alerts and/or colorful labeling, on the electronic or paper-based patient’s medical record, preventing future events from occurring with the same me …**		
a. Yes	180 (62.3)	0.56–0.68
b. No	109 (37.7)	0.32–0.44
**27. Which activities in the field of safe medication practice are implemented in your hospital on a regular basis (more than 50%)? (check all that apply)**		
a. Unit dose dispensing	280 (96.9)	0.94–0.99
b. Centralized cytotoxic preparation	220 (76.1)	0.71–0.81
c. Centralized intravenous administration service	220 (76.1)	0.71–0.81
d. Therapeutic drug monitoring	220 (76.1)	0.71–0.81
e. Drug information	260 (90)	0.86–0.93
f. Pharmacists round with physicians	140 (48.4)	0.43–0.54
g. Pharmacists round independent of physicians	80 (27.7)	0.22–0.33
h. Patient Counseling at Discharge	268 (92.7)	0.89–0.95
i. Medication reconciliation	260 (90)	0.86–0.93
j. ADEs reporting	260 (90)	0.86–0.93
k. SBAR communication	2 (0.7)	0.01–0.02
l. None of the above	1 (0.3)	0.01–0.02
**28. Which of the following medication incidents are encountered in your hospital? (check all that apply)**		
a. Wrong /unclear dose of strength of frequency	289 (100)	1
b. Wrong Dosage Form	53 (18.3)	0.14–0.23
c. Wrong medication	80 (27.7)	0.22–0.33
d. Wrong Route	99 (34.3)	0.29–0.40
e. Omitted/delayed medication	90 (31.1)	0.25–0.37
f. Wrong label	60 (20.8)	0.16–0.26
g. Wrong storage	84 (29.1)	0.24–0.35
h. Wrong Method Preparation	60 (20.8)	0.16–0.25
i. Passed expiry date	82 (28.4)	0.23–0.34
j. Contraindicated medication	70 (24.2)	0.19–0.30
k. Allergy to medication	90 (31.1)	0.25–0.37
l. Mismatching patients	70 (24.2)	0.19–0.30
**29. Which of the following causes have been behind medication incidents in your hospital? (check all that apply)**		
a. Breakdown or communication at transfer and hand-offs	30 (10.3)	0.07–0.14
b. Poor/improper documentation	60 (20.8)	0.16–0.26
c. Inaccurate dosage calculations	30 (10.3)	0.07–0.14
d. Unavailability of electronic system	40 (13.8)	0.10–0.18
e. No Written Policies and procedures	20 (6.9)	0.04–0.10
f. No/Insufficient trainings	30 (10.3)	0.07–0.14
g. High Workload Pressures	289 (100)	1
h. Insufficient Human Resources	20 (6.9)	0.04–0.10
i. Lapse in individual performance	30 (10.3)	0.07–0.14

Furthermore, pharmacists attested that 96.5% of adverse drug reactions are only reported when they are serious and life-threatening, or when they are severe and cause disability. Moreover, 96.5% stated that they would first report these adverse drug reactions to Saudi FDA. When questioned regarding the most preferred method used to report adverse drug reactions, nearly 70% preferred a formal letter/ email, and 96.5% preferred to do so via the national pharmacovigilance center. When asked about the educational activities they’ve attended, the pharmacists reported that83% did not attend a seminar or congress about medication safety in the past year, and 49.8% had never taken a pharmacovigilance workshop/course during their entire career. About 70% of the pharmacists declared reporting adverse drug events more than once a week. In addition, 98% of the participants agreed that medication safety programs should be introduced into the pharmacy university curriculums Table 4.

## Discussion

In this study, we aimed to assess the knowledge, attitude, and practice of medication safety of practicing pharmacists by using a representative sample of hospital pharmacists in Saudi Arabia. The majority of pharmacists knew about ADRs and PV concepts and PV function. These results were consistent with previous studies on the knowledge pertaining to the concepts of ADRs and PV [9, 16, 20–23]. However, the findings revealed inadequate knowledge about the overall PV discipline among study participants, where the majority of the pharmacists failed to correctly answer questions related to independent ADRs treatment, augmented drug reaction, the international location of ADR, and the WHO “online database” for reporting ADRs. Moreover, we found incomplete and/or wrong answers for the questions that included one or more correct answers. Similar findings were reported narrow knowledge of PV from previous studies [9, 24]. These are critical findings; as inadequate knowledge of PV is associated with a high degree of underreporting of ADRs [25, 26]. These findings were not surprising considering the fact that the Saudi PV system is in its infancy in comparison to developed countries that have advanced systems. This suggests that the national PV program should be supported by providing adequate training to pharmacists to ensure that the program meets its planned goals.

In the current study, the pharmacists had very positive attitudes toward PV and ADRs reporting. Attitudes of pharmacists were positive towards the importance of reporting ADRs, to enable safe drug practice, and that reporting ADRs should be made mandatory for practicing pharmacists. Pharmacists in the current study reported that pharmacists and doctors are the most qualified health care professionals to play the role of PV and ADRs reporting, which is in agreement with findings from other studies [9, 17, 21, 27]. Less than 50 % believed that it is a professional obligation to report ADRs. This finding was dissimilar to previous studies which agreed that reporting ADRs is a professional obligation [9, 17, 20, 28]. In fact, this finding is alarming and raises a question about its impact on precluding ADRs reporting. However, previous studies concluded that proper education and training in PV are determinant to improving attitudes, awareness, and ADR reporting rates.

A large percentage of surveyed pharmacists showed interest in participating in an ADR reporting system. In contrary to those beliefs, our findings showed that hospital staff pharmacists in Saudi Arabia were reluctant to report ADRs, which we hypothetically attribute to the fear of repercussion in the case of medical malpractice and the possible liability they may face. In fact, it is observed that event reporting mostly occurs in a “non-punitive milieu” where healthcare professionals are able to report any incidence without fear of repercussion or blame, a vital and indispensable step for improving medication safety [9, 29]. Moreover, the pharmacists have rated three major barriers that discourage their interest in reporting ADRs, including missing/unavailability of information about ADR, unaware of the need to report an ADR, and lack of time. Previous studies reported similar barriers that were also associated with ADRs underreporting [17, 20, 30–33]. Thus, it is clearly observed that pharmacists need to take on a preemptive role concerning the appraisal of the safety of a patient’s medication.

Moreover, the majority of the pharmacists asserted their hospitals have a medication safety committee, an established system for reporting ADEs, and a standardized form for reporting ADRs. These findings were expected as the majority of the Saudi hospitals have passed the Saudi CBAHI and JCI accreditations. Such accreditation bodies set structure standards and procedures to assure the provision of safe and quality care and services, including medication safety. Particularly, fulfilling the requirements of these accreditation bodies requires rigorous preparations and application of measurable elements of compliance with medication safety standards and procedures in terms of availability of ADRs reporting systems and evidence of staff training.

Data from this study showed that the majority of pharmacists (96.5%) would prefer using online reporting via the national PV center system and 69.6% preferred formal email/letter. This is in contrast to a previous study survey in which the pharmacists preferred reporting tools were via paper-based forms (33.3%), over phone calls (23.2%), and through using the Internet (4.3%) [17]. However, health care practitioners in Saudi Arabia can report ADRs via different and varied methods, such as online or mail, fax or phone, and postal mail [14]. The PV center at the SFDA is utilizing two different advanced electronic databases, the Empirica Trace and Vigibase belong to the WHO-UMC. The Vigibase helps to do data mining by the PV centers’ staff, and Empirica Trace is used for collecting, storing, analyzing, and evaluating the reports received from health care professionals, pharmaceutical companies, and patients [14].

About half of the pharmacists in this study have encountered ADRs during their practice. Prior studies showed that ADRs identification varies significantly by pharmacists, ranging from less than 20% to over 65% [9, 16, 23, 27, 32]. Some pharmacists did not know where to report ADRs, which is considered as a major barrier in participation in PV. However, surveyed pharmacists thought ADRs should be reported to patients, prescribers, drug companies, and Saudi MOH. It is imperative for hospitals to teach pharmacists that ADRs should be reported to the official regulatory authority of the PV center at the SFDA, because SFDA is an integral entity of the Saudi health care system, even though it is operated by an independent administration. Previous studies from other countries also reported health care professionals’ confusion with the reporting channel of ADRs [20, 23, 33].

The pharmacists in this study have indicated that ADRs are reported more than once a week in their workplace. Indeed, all pharmacists revealed that wrong /unclear dose of the strength of frequency as the most encountered medication incidents which is contributed to a high workload. These findings correspond with previous studies reporting overworked health care professionals as a cause of ME [34, 35]. Potential strategies have been explored to reduce ME and improve medication safety like medication reviews [36] and reconciliation [37], automated information systems [38–40], education [41], or employing multi-component interventions [42].

We found that 83% of pharmacists did not attend any continuing education program on safe medication practice issues in the last year and almost half of them never attended any course or workshop about PV. However, the majority agreed that medication safety programs should be included in the actual pharmacy curriculum and PV should be taught in detail to healthcare professionals. Given the importance of PV in understanding and preventing ADEs and ADR reporting, strengthening the continuous training and education in the fields of PV and ADRs is needed to improve the safety and quality of patients’ life. Relevant authorities and hospitals, should pursue and initiate a continuous mandatory education program on PV and/or ADR reporting for health care professionals across Saudi Arabia. The WHO and the International Society of Pharmacovigilance had issued the core component of a comprehensive PV curriculum to support the PV integration into the curriculums of healthcare schools, including pharmacy to improve their knowledge about PV [43].

There are some limitations to this current study. The study relied on the pharmacists’ own self-assessment of their knowledge, attitudes, and practice towards PV, which might be considered social desirability bias since some participants may be unwilling to reveal practice deficiencies. Thus the real knowledge, attitudes, and practice of pharmacists cannot be correctly generalized. The questionnaire was administered to hospital pharmacists and as such, the results cannot be generalized to pharmacists working in other settings, such as community, primary, secondary pharmacies. Also, we have not conducted bivariate and multivariate analyses, therefore, we did not stratify the differences between the pharmacists’ positions and hospital types. Finally, social desirability bias.

## Conclusion

The present study highlighted a narrow of knowledge in the discipline of PV and ADR reporting among pharmacists working in Riyadh hospitals. A positive attitude and a satisfactory practice was observed amongst surveyed pharmacists toward PV and ADRs reporting. The topmost reported barriers to reporting ADRs were missing/unavailability of information about ADRs, lack of awareness of the need to report an ADRs, and lack of time. Establishing formal and frequent educational programs and re-enforcement and surveillance plans for pharmacists, will promote the understanding of the ADRs reporting process and requirements and will improve the PV system in hospitals.

## Supplementary Information

**Additional file 1.**

## Data Availability

The datasets used and/or analyzed during the current study are available from the corresponding author on reasonable request.

## Abbreviations

PV: Pharmacovigilance
ADR: Adverse drug reactions
ME: Medication errors
ADEs: Adverse drug events
WHO: World Health Organization (WHO)
UMC: Uppsala Monitoring Centre
SFDA: Saudi Food and Drug Administration
CBAHI: Central Board for Accreditation of Healthcare Institutes
JCI: Joint Commission International
ISO: International Organization for Standardization
