# Supplementary material for: Medication safety knowledge, attitude, and practice among hospital pharmacists in tertiary care hospitals in Saudi Arabia: a multi-center study
Source: Arch Public Health. 2021 Jul 12;79:130. doi: 10.1186/s13690-021-00616-1 (PMC8274029; doi:10.1186/s13690-021-00616-1)
Supplement: Supplementary file 1 — Additional file 1. [file 13690_2021_616_MOESM1_ESM.doc]

***Medication safety knowledge, attitude, and practice among hospital pharmacists in Tertiary Care Hospitals in Saudi Arabia: A multi -center study***

You are receiving this questionnaire to evaluate the Knowledge, Attitude, and Practice of medication safety among hospital pharmacists in Saudi Arabia. The aim of this survey is to assess the educational needs of hospital pharmacists in relevance to

pharmacovigilance and adverse events reporting, and enhance medication safety culture in Saudi Arabia. Your feedback is vital to the process. We appreciate the time allocated to complete the following survey which shall take around 30 minutes of your time. Your responses will be confidential and anonymous. All responses will be compiled together and analyzed as a group.

**Abbreviations: KAP**: *Knowledge, Assessment, Practice*; **ADR**: *Adverse Drug Reaction*.

***Hospital: A. Pharmacist's Profile***

1. **Gender:**
   1. Male
   2. Female
2. **Age Group (years):**
   1. 21-30
   2. 31-40
   3. >40
3. **Educational Qualification(s) (kindly check all that apply):**
   1. BSc in Pharmacy
   2. PharmD
   3. Masters
4. **Professional Status:**
   1. Pharmacist Assistant
   2. Staff Pharmacist
   3. Clinical Pharmacist
   4. Senior Pharmacist
5. **Experience (years):**
   1. < 5
   2. 5 - 10
   3. > 10

***B. Hospital's Description***:

1. **Business Structure**
   1. Public
   2. Private
   3. University Medical Center
2. **Number of Beds:**
   1. ≤50
   2. 51 - 100
   3. 101 - 299
   4. ≥300
3. **Occupancy Rate:**
   1. <50%.
   2. 50% - 65%
   3. 66% - 80%
   4. >80%
4. **Accreditation or Certification**
   1. CBAHI Accredited
   2. JCI Accredited
   3. ISO Certified
   4. Magnet Certified
   5. Not Accredited / In process

***KAP Questions:***

*Section A: includes 19 questions related to basic knowledge and information on medication safety.*

*Section B: Includes 15 questions related to hospital pharmacist's attitude.*

*Section C: Includes 29 questions related to practice regarding identification, reporting system, and analysis, and prevention of adverse drug events.*

***Section A: Knowledge* (Correct answers)**

*Choose one answer for question 1 to 19.*

1. **A side Effect is an Adverse Drug Reaction:**
   1. Yes
   2. No
2. **An Adverse Drug Reaction is:**
   1. Preventable
   2. Non-preventable
   3. Preventable and non-preventable
3. **Do you believe all drugs available in the market are safe?**
   1. Yes
   2. No
4. **Which of the following is a Medication Safety Agency?**
   1. Institute for Safe Medication Practices (ISMP)
   2. Uppsala Monitoring Center (UMC)
   3. Agency for Healthcare Research and Quality (AHRQ)
   4. European Medicines Agency (EMA)
   5. All of the above
   6. None of the above
5. **Have you ever heard about the concept of Pharmacovigilance?**
   1. Yes
   2. No
6. **Pharmacovigilance is the study that relates to:**
   1. Safe, effective, appropriate and economic use of medicines
   2. Therapeutic drug monitoring
   3. Detection, assessment, understanding & prevention of adverse effects
   4. All of the above
7. **The functions of Pharmacovigilance are:**
   1. Detection and study of ADRs
   2. Measurement of risk and effectiveness of drug use
   3. Dissemination of ADR information and education
   4. All of the above
8. **Pharmacovigilance concerns:**
   1. Drug related problems
   2. Herbal products
   3. Medical devices and vaccines
   4. All of the above
9. **Pharmacovigilance consists of:**
   1. ADRs
   2. ADEs
   3. MEs
   4. All of the above
10. **ADRs which are independent can be treated by:**
    1. Withdrawing the drug
    2. Reducing the dose
    3. Replacing the medication
    4. All of the above
11. **Augmented drug reaction is:**
    1. Dose dependent, common in occurrence, rarely fatal
    2. Dose independent, comparatively rare in occurrence, more fatal
    3. a & b
    4. None of the above
12. **The International center of ADR monitoring is located in:**
    1. United States of America
    2. France
    3. Australia
    4. Sweden
    5. Don't know
13. **Which of the followings is the "WHO online database" for reporting ADRs:**
    1. ADR advisory committee
    2. Medsafe
    3. Eudra Vigilance
    4. VigiBase
    5. MedWatch
    6. None of the above
14. **Is there a National Pharmacovigilance Center (ADR reporting center) in Saudi Arabia:**
    1. Yes
    2. No
    3. Don't know

*Choose one or multiple answers for questions 15 to 19.*

1. **A never event:**
   1. is a preventable incident causes serious harm
   2. is a medication and non-medication related-event
   3. is a medication-related event only
   4. Don't know
2. **A sentinel event:**
   1. is an unexpected incident
   2. causes death or permanent disability
   3. needs immediate investigation and attention
   4. is a medication and non-medication related event?
   5. is a medication-related event only
   6. Don't know
3. **Root Cause Analysis (RCA):**
   1. is a prospective risk assessment method
   2. is a retrospective risk assessment method
   3. identifies and prevents problems after they occur
   4. examines possible process and system failures
   5. provides the underlying cause
   6. develops corrective actions and preventive actions
4. **"Just Culture":**
   1. is a non-punitive or blame-free culture
   2. is an open and fair culture
   3. develops accountability towards staff's actions
   4. cultivates trust in workplace
   5. encourages people to speak up about mistakes
   6. increases reporting of medication errors
   7. decreases the incidence of medication errors
5. **A Failure Mode and Effects Analysis (FMEA):**
   1. is a prospective risk assessment method?
   2. is a retrospective risk assessment method?
   3. identified and prevents problems before they occur
   4. examines possible process and/or product failures
   5. provides the anticipated result
   6. develops corrective actions and preventive actions

***Section B: Attitude***

1. **Which of the following health care professionals is/are responsible for ADRs reporting in your hospital (kindly check all that apply):**
   1. Doctor
   2. Pharmacist
   3. Nurses

*Choose one answer for questions 2 to 11.*

1. **Do you support "direct ADR reporting" by the patient instead of health care professionals?**
   1. Yes
   2. No
2. **Do you think that ADR reporting and monitoring system would benefit the patient?**
   1. Yes
   2. No

1. **Do you think pharmacists are the ones to assist physicians in ADR reporting?**
   1. Yes
   2. No
2. **Do you worry about legal problems while you think of ADR?**
   1. Yes
   2. No
3. **Do you feel that ADR reporting is time consuming activity with no outcome?**
   1. Yes
   2. No
4. **Do you think reporting is a professional obligation to you:**
   1. Yes
   2. No
   3. Don't know
   4. Perhaps
5. **Do you believe reporting should be made mandatory for practicing pharmacist?**
   1. Yes
   2. No
   3. Don't know
6. **Are you interested in participating in an ADR reposting system?**
   1. Yes
   2. No
7. **Do you think there should be a National Pharmacovigilance Program?**
   1. Yes
   2. No
8. **If a National Pharmacovigilance Program is instituted, what is your expectation from it?**
   1. Someone from the center to coordinate with you
   2. Financial compensation for time and energy spent

*Choose one or multiple answer for questions 12 to 15.*

1. **ADR reposting is important in order to:**
   1. enable safe drugs to be used
   2. measure the incidence of ADRs
   3. identify factors that might predispose to an ADR
   4. identify previously unrecognized ADRs
   5. compare ADRs for drugs in similar therapeutic classes
   6. compare ADRs of the same drug from different drug companies
2. **In your opinion, pharmacist is encouraged to report ADRs when:**
   1. the reaction is of a serious nature
   2. the reaction is unusual
   3. the reaction is to a new product
   4. the reaction is not reported before a particular drug
   5. the reaction is well recognized for a particular drug
3. **In your opinion, pharmacists may be discouraged to report ADRs when:**
   1. Level of clinical knowledge makes it difficult to decide whether or not an ADR has occurred
   2. Uncertain association between the drug and the adverse reaction
   3. The ADR is too trivial to report
   4. Concern that a report will generate extra work
   5. ADR reporting from is not available when needed
   6. No enough information available from the patient
   7. Lack of time to fill in a report
   8. Did not know how to report
   9. Unaware of the need to report an

ADR

- 1. Consider if the doctors' responsibility
  2. Fear of legal liability

1. **The perception of a safety culture revolves around which of the following statements (check one response):**
   1. Why waste our time on safety
   2. We do something when we have an incident
   3. We have systems in place to manage all identified risks
   4. We are always on the alert for risks that might emerge
   5. Risk management is an integral part of everything that we do

***Section C: Practice*** *(Chose one or* *multiple answers for question 1 to 29).*

1. **Concerning the national accreditation Pharmacy chapter, are all medication safety related standards applied in your hospital?**
   1. Yes
   2. No
2. **Does your hospital have written policies and procedures on safe medication practice?**
   1. Yes
   2. No
3. **Has your hospital established a defined system for adverse events reporting?**
   1. Yes
   2. No
4. **Is there a "Medication Safety" or a "Safety Committee/Department" in your hospital?**
   1. Yes
   2. No
5. **Is yes, is the pharmacist a member of the committee/department?**
   1. Yes
   2. No
6. **If yes, does the pharmacist chair/oversee that committee/department?**
   1. Yes
   2. No
7. **In your hospital, is there a standardized form for reporting ADRs?**
   1. Yes
   2. No
8. **Is the reporting form available at your Pharmacy?**
   1. Yes
   2. No
9. **Does your workplace encourage you to report any ADR?**
   1. Yes
   2. No
10. **Have you ever come across with an ADR?**
    1. Yes
    2. No
11. **In your hospital, ADRs are reported only when they are:**
    1. Serious and life-threatening
    2. Severe and cause disability
    3. Mild and cause less inconvenience
12. **When an ADR is encountered in your hospital, it is reported to:**
    1. Patient
    2. Prescriber
    3. Drug Company
    4. OPL Drug Information Center
    5. MOPH
    6. Saudi FDA.
13. **How do you prefer to report ADRs to drug companies?**
    1. Verbally inform the representative of the drug company on routine visits
    2. Formal e-mail/letter
    3. Phone call
    4. via a National Pharmacovigilance Center
14. **Have you attended any congress/ seminar on continuing educational program on safe medication practice issues in the last year?**
    1. Yes
    2. No
15. **Have you ever had a course/attended a workshop about pharmacovigilance?**
    1. Yes
    2. No
16. **Have you anytime read any article on prevention of ADRs?**
    1. Yes
    2. No
17. **Have you ever been trained on how to report ADRs?**
    1. Yes
    2. No
18. **Do you think Pharmacovigilance should be taught in detail to healthcare professionals?**
    1. Yes
    2. No
19. **Do you think medication safety (ADR) programs should be included in the actual Pharmacy curriculum?**
    1. Yes
    2. No
    3. Not necessary

1. **Which of the following medication safety preventive measures are applied in your Pharmacy?**
   1. Unit dose labeling
   2. Unit dose labeling per patient
   3. Look-A-Like / Sound-A-Like labeling
   4. High Alert Medication labeling
   5. "Store in Fridge" labeling
   6. Use of TALLman letters
   7. Avoidance of ambiguous nomenclature (abbreviations, trailing zeroes)
   8. Bar-coding
   9. Temperature monitoring
2. **In your Pharmacy, are there any staff educational sessions on medication safety best practices?**
   1. Yes
   2. No
3. **When ADRs are reported, which of the following assessment methods are implemented in your hospital:**
   1. Root Cause Analyses (RCA)
   2. Failure Mode and Effects Analysis (FMEA)
   3. Causality Assessment tools
   4. Severity Assessment tools
   5. Classification tools
   6. All of the above
   7. None of the above
4. **Are analysis results reported to the Pharmacy & Therapeutics committee?**
   1. Yes
   2. No
5. **How often are ADRs reported?**
   1. More than once a week
   2. Once a month
   3. A few times a year
   4. Never
6. **Whether electronic and/or paper-based, are ADRs documented in the patient medical record?**
   1. Yes
   2. No
7. **If yes, is there an alerting system, such as pop-up alerts and/pr colorful labeling, on the electronic or paper-based patient's medical record, preventing future events from occurring with the*.***

same medication?

- 1. Yes
  2. No

1. **Which activities in the field of safe medication practice are implemented in your hospital on regular basis (more than 50%)?**
   1. Unit dose dispensing
   2. Centralized cytotoxic preparation
   3. Centralized intravenous administration service
   4. Therapeutic drug monitoring
   5. Drug information
   6. Pharmacists round with physicians
   7. Pharmacists round independent of physicians
   8. Patient counseling at discharge
   9. Medication reconciliation
   10. ADEs reporting
   11. SBAR communication
   12. None of the above
2. **Which of the following medication incidents are encountered in your hospital?**
   1. Wrong /unclear dose of strength of frequency
   2. Wrong dosage form
   3. Wrong medication
   4. Wrong route
   5. Omitted/delayed medication
   6. Wrong label
   7. Wrong storage
   8. Wrong method of preparation
   9. Passed expiry date
   10. Contra-indicated medication
   11. Allergy to medication
   12. Mismatching patients
3. **Which of the following causes have been behind medication incidents in your hospital?**
   1. Breakdown or communication at transfer and hand-offs
   2. Poor/improper documentation
   3. Inaccurate dosage calculations
   4. Unavailability of electronic system
   5. No written policies and procedures
   6. No/Insufficient trainings
   7. High workload pressures
   8. Insufficient human resources
   9. Lapse in individual performance
